# Protein turnover and differentiation in *Leishmania*

**DOI:** 10.1016/j.ijpara.2007.03.008

**Published:** 2007-08

**Authors:** Sébastien Besteiro, Roderick A.M. Williams, Graham H. Coombs, Jeremy C. Mottram

**Affiliations:** a Wellcome Centre for Molecular Parasitology and Division of Infection & Immunity, Institute of Biomedical and Life Sciences, University of Glasgow, 120 University Place, Glasgow G12 8TA, UK; b Strathclyde Institute of Pharmacy and Biomedical Sciences, University of Strathclyde, Glasgow G4 0NR, UK

**Keywords:** *Leishmania*, Differentiation, Protease, Peptidase, Genome

## Abstract

*Leishmania* occurs in several developmental forms and thus undergoes complex cell differentiation events during its life-cycle. Those are required to allow the parasite to adapt to the different environmental conditions. The sequencing of the genome of *L. major* has facilitated the identification of the parasite’s vast arsenal of proteolytic enzymes, a few of which have already been carefully studied and found to be important for the development and virulence of the parasite. This review focuses on these peptidases and their role in the cellular differentiation of *Leishmania* through their key involvement in a variety of degradative pathways in the lysosomal and autophagy networks.

## 1 Introduction

**Fig. 1:**
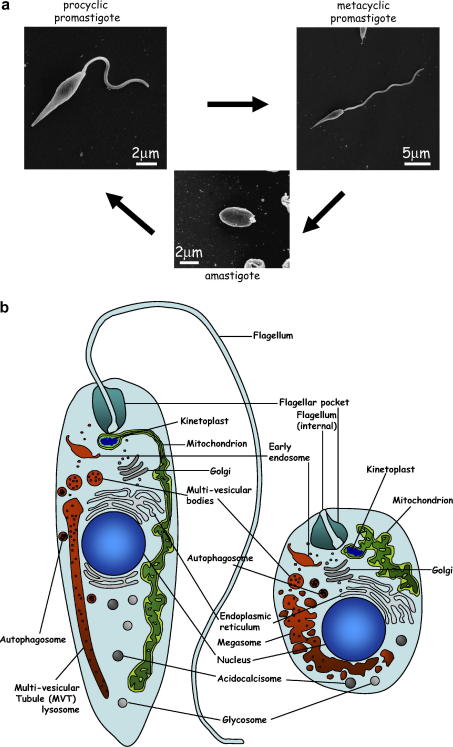
Changes in cell shape during the *Leishmania* life-cycle. (a) Scanning electron microscope images of the main *Leishmania major* life-cycle stages, the procyclic and metacyclic promastigotes were grown in culture, the amastigote was isolated from an infected macrophage isolated from a mouse. (b) Schematic representation of the main intracellular organelles from *Leishmania* promastigote (left) or amastigote (right) forms. The flagellar pocket marks the anterior end of the cell.

*Leishmania* are protozoan parasites with a complex life-cycle, involving several developmental forms (Fig. 1a). These forms represent an adaptation to the changing environmental conditions encountered by the parasites within their two hosts: the mammalian host, to which they are pathogenic, and the sandfly insect vector. In the sandfly, *Leishmania* replicate as extracellular and actively motile flagellated cells known as promastigotes (Fig. 1b, left), which reside primarily in the insect’s alimentary tract. Two main forms can be distinguished (although several other intermediate forms have been reported (Bates and Rogers, 2004; Gossage et al., 2003)): multiplicative, but not mammalian-infective, procyclic promastigotes that are present in the insect’s midgut; non-dividing, but mammalian-infective, metacyclic promastigotes in the thoracic midgut and proboscis of the sandfly. The metacyclic promastigotes, when inoculated into a mammalian host through a sandfly bite, differentiate (after being phagocytosed by a macrophage) into the intracellular aflagellate amastigote form (Fig. 1b, right). This form of the parasite resides within a vacuole with lysosomal features that is termed the parasitophorous vacuole.

During transition through these different extra- and intracellular environments, *Leishmania* are exposed to many changes in their living conditions: for example, there are variations in the availability and type of nutrients, pH, temperature, as well as the availability of oxygen. The strategy adopted by the parasites to survive these changes is to develop into highly specialised and adapted forms. These developmental forms are distinguished by their nutritional requirements, their growth rate and ability to divide, the regulated expression of their surface molecules, and also their morphology. Metacyclic promastigotes are different from the procyclic forms in that they are pre-adapted for survival in the mammalian host: for instance, they express stage-specific surface molecules and become complement-resistant. Amastigotes multiply within the parasitophorous vacuole in macrophages and are highly adapted morphologically to this compartment: as they are intracellular, non-motile forms, they have a reduced size and have a much-reduced flagellum that does not emerge from the flagellar pocket (Fig. 1b, right). They are also acidophiles, adapted to the low pH of this compartment, and have an adapted energy metabolism.

The two differentiation events mainly studied with *Leishmania* are the procyclic to metacyclic differentiation of promastigotes (also called metacyclogenesis) and the metacyclic promastigote to amastigote transformation inside the host macrophage. Some factors triggering these events in vitro have been characterised. For instance, low pH, lack of oxygen and nutritional depletion of tetrahydrobiopterin can trigger metacyclogenesis. Conditions mimicking a phagolysosome-like environment, such as low pH, a temperature of 37 °C and elevated CO_2_, can induce the promastigote to amastigote differentiation (Barak et al., 2005). Although, these environmental factors triggering *Leishmania* differentiation in vitro were recognised several years ago, relatively little is known about the molecular processes that mediate the cellular remodelling. It is likely that a series of changes in gene expression are instrumental in the morphological changes associated with differentiation to the individual developmental forms. However, in *Leishmania* protein-coding genes are transcribed as polycistronic RNAs and they are apparently not regulated at a transcriptional level (Campbell et al., 2003), which makes the identification of stage-specific genes problematic. Recent transcriptomic and proteomic approaches to identify stage-regulated genes and proteins are promising, but the studies have been carried out on different *Leishmania* species and are therefore difficult to compare (Holzer et al., 2006; McNicoll et al., 2006; Saxena et al., 2007; Walker et al., 2006). Some of the most clear-cut stage-specific markers include peptidases, some of which have been known to be associated with the mammalian virulence of *Leishmania* for a long time (Mottram et al., 2004), and whose functions range from nutrient acquisition to cellular reshaping and recycling (Mottram et al., 2004; Williams et al., 2006). Thus these peptidases, and probably others too, are instrumental to the differentiation of the parasite. Their involvement in these processes is the focus of this review.

## 2 The degradative capacity of *Leishmania major*

**Fig. 2:**
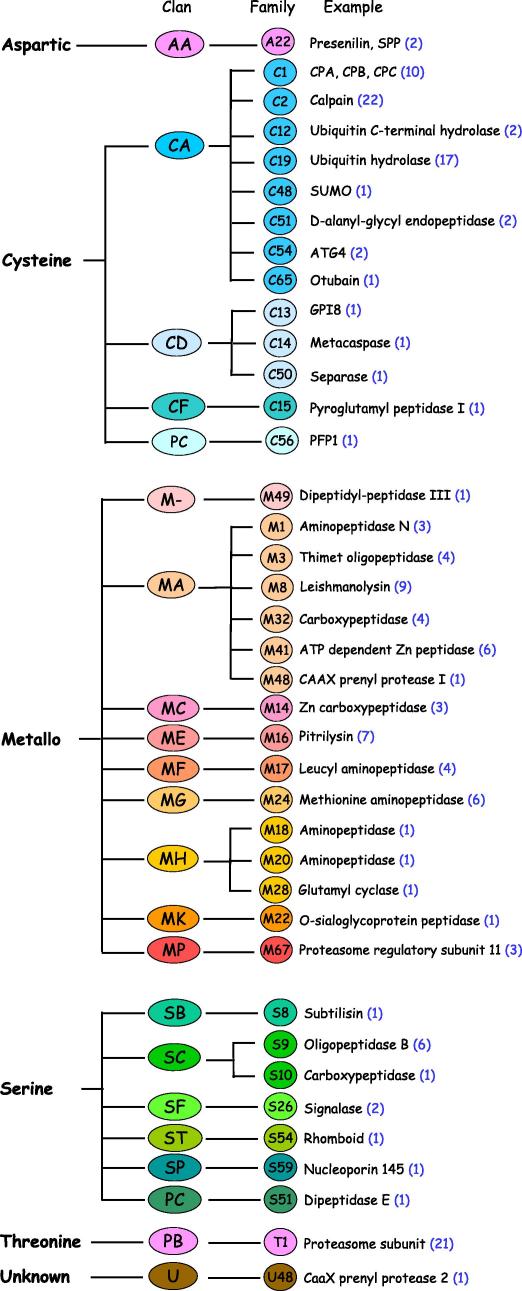
Clans and families of *Leishmania major* peptidases. Nomenclature is based on the MEROPS database (http://merops.sanger.ac.uk/). Numbers in brackets represents the estimated number of peptidases in each Family, taken from Ivens et al. (2005) and the MEROPS database (release 7.7, January 2007).

Peptidases are a structurally and functionally diverse set of enzymes that hydrolyse proteins and they can be grouped into distinct Clans and Families based on intrinsic evolutionary relationships (see the MEROPS database; Rawlings et al., 2004b). A starting point for an analysis of protein turnover in *Leishmania* is an evaluation of the complete complement of peptidases in the parasite. This was first carried out for *Leishmania major* as part of the genome analysis (Ivens et al., 2005), but has been updated in this review to reflect recent changes in nomenclature in the MEROPS database. *Leishmania major* was predicted to contain at least 154 peptidases (including aspartic-, cysteine-, metallo-, serine- and threonine-peptidases (Table 1 and Fig. 2)), representing ∼1.8% of the genome. Thus this set of peptidases is more complex than those of *Saccharomyces cerevisiae* and *Plasmodium falciparum*, but considerably less complex than that of mammals (Table 1). The findings for *Leishmania* were subsequently confirmed when the genomes of *Leishmania infantum* and *Leishmania braziliensis* were fully sequenced (http://merops.sanger.ac.uk/), showing that all three species have a very similar array of peptidases. Such database searches are extremely informative in predicting, based on homologies, gene contents, but it should not be discounted that *Leishmania* contains as yet unidentified peptidases that have roles in the cell that have not yet been described in other organisms.

### 2.1 Aspartic peptidases and metallopeptidases

*Leishmania major* has just two aspartic peptidases. One has sequence similarity to presenilin 1 (PS1) which is a multi-pass membrane peptidase that cleaves type I membrane proteins, such as the amyloid precursor protein of Alzheimer’s disease and the Notch receptor that is involved in signalling during differentiation and development (Xia and Wolfe, 2003). There is also the suggestion that PS1 has a role in autophagy (see Section 4). The second is an enzyme that has sequence identity to an intramembrane signal peptide peptidase (SPP) that cleaves signal peptides within their transmembrane region. Whilst SPP appears to be ubiquitous in eukaryotes, PS1-like peptidases have been described in mammals, worms and *Dictyostelium*, but not detected in yeast or other protists such as *Plasmodium*. In contrast, pepsin-like aspartic peptidases such as the plasmepsins, which are so abundant in *Plasmodium* and other apicomplexan parasites (Coombs et al., 2001), are apparently entirely absent from *Leishmania*.

There are 16 families of metallopeptidases represented in *L. major*, including a dipeptidyl-peptidase III homologue (family M49) that appears to be absent from trypanosomes. Metallopeptidases include many aminopeptidases, carboxypeptidases and dipeptidases, very few of which have been studied in *Leishmania*. One exception is leishmanolysin (also known as gp63 or MSP). *Leishmania* has multiple members of this gene family, which is the major glycosyl phosphatidyl inositol (GPI)-anchored surface protein and is thought to have a role in the parasite’s virulence and pathogenesis (Yao et al., 2003).

### 2.2 Cysteine peptidases

*Leishmania major* contains many distinct cysteine peptidase genes (Fig. 2). Papain family enzymes (Clan CA, family C1) have been extensively characterised in various *Leishmania* species, which contain cathepsin L-like enzymes (CPA and CPB) as well as cathepsin B-like enzymes (CPC) (Mottram et al., 2004). *CPB* occurs in a tandem array of eight similar genes in *L. major*, but the copy number of *CPB* genes and their polymorphism varies considerably between different species (Hide et al., 2007).

*Leishmania major* also contains many other Clan CA cysteine peptidases. Those with calpain domains are particularly abundant (22 genes), in distinct contrast to *S. cerevisiae* which has just a single gene. In higher eukaryotes, calpains play important roles in calcium-regulated functions such as signal transduction, cell differentiation and in apoptosis/necrosis, but little is known about their role in *L. major*. Another abundant group of cysteine peptidases are involved in ubiquination; the ubiquitin C-terminal hydrolases (19 genes), small ubiquitin-like modifier (SUMO)-specific peptidase (one gene) and otubain (one gene). A typical eukaryotic 20S proteasome has been characterised in *L. major* (Robertson, 1999) and genes encoding seven α- and 13β-threonine peptidase subunits of the proteasome can be identified. The presence of a fully functional proteasome in *L. major*, coupled to the presence of many ubiquitin and ubiquination enzymes, indicates that cytosolic protein degradation is important to the parasite. The precise function of this in differentiation of *Leishmania* deserves, and requires, further exploration. Several other predicted proteins of *L. major* have CHAP (cysteine, histidine-dependent amidohydrolases/peptidases) domains and might encode cysteine peptidases of the C51 family of d-alanyl-glycyl endopeptidases or peptidoglycan amidases. These are enzymes known to have roles in bacterial cell division, growth and cell lysis (Bateman and Rawlings, 2003), but have not yet been investigated in *Leishmania*. *Leishmania major* has two ATG4 cysteine peptidases (family C54), which play a central role in the autophagic pathway for turnover of proteins (see Section 4.1).

There are three Clan CD cysteine peptidases in *L. major*, GPI8, metacaspase and separase (Mottram et al., 2003). GPI8 is the catalytic subunit of the GPI:protein transamidase complex that attaches in the endoplasmic reticulum pre-formed GPI anchors onto precursor GPI-anchored proteins. In *Leishmania mexicana* the gene is dispensable and GPI8-deficient mutants are capable of differentiating into replicating amastigotes within macrophages in vitro and mice in vivo (Hilley et al., 2000). Metacaspases are evolutionary distant orthologues of metazoan caspases and appear to be restricted to plants, fungi and protozoa (Uren et al., 2000). They are cysteine peptidases of family C14 of Clan CD, with a catalytic cysteine and histidine dyad essential for enzyme activity. Plant and yeast metacaspases are thought to play a crucial role in the induction of programmed cell death (del Pozo and Lam, 1998; Herker et al., 2004; Madeo et al., 2002) and the *L. major* metacaspase, which has an arginine substrate specificity, can complement an apoptosis cell death phenotype in yeast (Gonzales et al., 2007). A role in programmed cell death, however, has not been demonstrated in *Leishmania*, rather the metacaspase seems to be primarily involved in cell cycle events (Ambit, Fasel, Coombs and Mottram, unpublished data). Separase is a key regulator of the metaphase-to-anaphase transition in higher eukaryotes (Yanagida, 2005) and whilst little is known about chromosome segregation in *Leishmania*, separase is likely to have a crucial role in the parasite’s cell cycle control mechanisms.

Pyroglutamyl peptidase I (PPI) is a cysteine peptidase of family C15 of the Clan CF that removes N-terminal l-pyroglutamyl residues (l-pGlu). The l-pGlu modification is a post-transcriptional modification that confers relative aminopeptidase resistance and, in some cases, is essential to the modified peptides’ biological activity. An *L. major* homologue (*LmjPPI*) has been cloned and recombinant PPI had pyroglutamyl peptidase activity. *LmjPPI* null mutants were differentiation competent and could establish infections in a mouse infection model (Schaeffer et al., 2006). However, whilst PPI is not essential for normal cell function it could be involved in regulating the action of l-pGlu-modified peptides required for differentiation of *L. major* (Schaeffer et al., 2006), as overexpression of active, but not a catalytically dead, PPI caused a metacyclogenesis defect. PFPI is a multimeric cysteine peptidase from *Pyrococcus furiosus*, and is the enzyme-type of family C56 of Clan PC. Genome analyses indicate that orthologues are present in rather few other organisms, including several bacteria, archaea and plants. A PFP1-like protein is expressed in *L. majo*r, but not other species of *Leishmania* or trypanosomes (Eschenlauer et al., 2006). The expression of *PFP1* in *L. major* suggests that PFP1 might contribute to the disease tropism that distinguishes this *Leishmania* species from others.

### 2.3 Serine peptidases

One of the most abundant group of serine peptidases in metazoa are the trypsin/chymotrypsin family (S01). However, no members of this family appear to be present in *Leishmania*, although there are representatives of seven other serine peptidase families. There is one subtilisin-like serine peptidase and the presence of a predicted signal peptide suggests that it might reside in the secretory/endosomal system. *Leishmania major* has six genes that encode proteins predicted to belong to the S9 family, including prolyloligopeptidase (POP), peptidyl-dipeptidase IV and oligopeptidase B (OPB), which occurs only in plants, bacteria and kinetoplastids. All these enzymes contain a characteristic aspartate, histidine, serine catalytic triad of residues, are moderately sized (∼80 kDa) multidomain proteins, with a globular catalytic domain containing a large active site cleft and a β-propeller domain that limits access to the active site. OPB and POP have been shown to be important in the virulence of *Trypanosoma cruzi*, mediating entry into host cells (Caler et al., 1998). Inhibitors of parasite POP prevent host cell invasion (Grellier et al., 2001). *Trypanosoma brucei* OPB is released into the serum of infected hosts, and mediates host damage (Morty et al., 2005). Other serine peptidases apparently present in *L. major* include a lysosomal serine carboxypeptidase (Parussini et al., 2003), a type I signal peptide peptidase, which cleaves signal peptides from proteins in the endoplasmic reticulum that have entered the secretory pathway, a 26S regulatory subunit of the proteasome, and a nucleoporin homologue. *Leishmania major* also has a gene encoding a rhomboid-like protein. These are intramembrane serine peptidases conserved in eukaryotes and prokaryotes with divergent biological functions that include quorum sensing in bacteria, mitochondrial membrane fusion, apoptosis and stem cell differentiation (Urban, 2006). The presence of a mitochondrial-like N-terminal targeting sequence suggests that the *L. major* rhomboid might have a mitochondrial function.

### 2.4 Peptidase inhibitors

There are a large number of peptidases encoded in the *Leishmania* genome, yet little is known about the roles of many of them, nor how their activities are regulated in order to fulfil their function. The activities of some peptidases are controlled by proteinaceous inhibitors which they encounter in their natural environment. The human genome contains an abundance (183) of genes encoding such natural peptidase inhibitors, such as the serpins (inhibitors of serine peptidases) and cystatins (inhibitors of cysteine peptidases) (Rawlings et al., 2004a). Although, *L. major* expresses large amounts of peptidases there are no genes encoding cystatin- or serpin-like proteins in its genome (Ivens et al., 2005). *Leishmania major* does contain a tight-binding reversible cysteine peptidase inhibitor (ICP) that otherwise occurs in just a few lower eukaryotes and some bacteria (Rigden et al., 2002; Sanderson et al., 2003). The *L. mexicana* ICP is a potent inhibitor of Family C1 peptidases, such as mammalian cathepsin-l and CPB (Sanderson et al., 2003) and the *L. mexicana* ICP has been postulated to play a role in the host–parasite interaction rather than in the control of endogenous parasite enzymes (Besteiro et al., 2004). The solution structure of *L. mexicana* ICP has been resolved and this showed that ICP has an immunoglobulin-like fold with three exposed loops, predicted to make interactions with the target enzymes (Smith et al., 2006). *Leishmania* also contain a second class of peptidase inhibitor, designated inhibitor of serine peptidase (ISP), which is similar in sequence to ecotin of *Escherichia coli* (Ivens et al., 2005). Ecotin of *E. coli* is a strong competitive inhibitor of trypsin-like serine peptidases (Chung et al., 1983), but these enzymes are not encoded in the *L. major* genome (Ivens et al., 2005), so it is likely that host-derived serine peptidases are the major target for the three ISPs that have been identified in the parasite. Candidate serine peptidases include chymotrypsin-like enzymes in the gut of the sandfly vector (Ramalho-Ortigao et al., 2003; Yan et al., 2001), or a variety of mammalian serine peptidases. Postulated in vivo targets of bacterial ecotin include serine peptidases expressed by cells of the innate immune system (i.e., neutrophils, mast cells, macrophages), such as neutrophil elastase (NE), tryptase and cathepsin G (CG), as well as enzymes participating in the coagulation cascades (Eggers et al., 2004). Activated neutrophils release serine peptidases together with chromatin fibers forming extracellular traps that disarm pathogens and play a role in killing bacteria (Brinkmann et al., 2004) and ecotin protects *E. coli* from killing by neutrophils, primarily due to the inhibition of neutrophil elastase (Eggers et al., 2004). A similar role for *Leishmania* ISP seems likely, especially as neutrophils have been postulated to be the first host cells of *Leishmania* upon inoculation into a mammal (Ribeiro-Gomes et al., 2004; Van Zandbergen et al., 2004).

Thus the peptidase complement of *Leishmania* is reasonably complex and has its own unique composition, presumably an adaptation to the unique life style of the parasite. At this stage, little is known about the functions in *Leishmania* of many of the enzymes. Nevertheless, some processes of protein turnover have been analysed and the peptidases involved have been characterised in part. The remainder of this review will focus on current knowledge on the occurrence and mechanisms of protein turnover in *Leishmania* and the role that this plays in the differentiation of the parasite.

## 3 Lysosomal peptidases and differentiation

*Leishmania* promastigotes are different from amastigotes in cell size and shape: procyclic promastigotes are spindle-shaped flagellated cells of about 20 μm cell body length, metacyclic promastigotes are also spindle shaped but shorter and with a relatively longer flagellum, whereas amastigotes are smaller (about 4-μm long), oblong and only retain a flagellar remnant within the flagellar pocket (Fig. 1b). However, similar components of the endocytic-lysosomal and exocytosis pathways are present in all forms, although there are differences in abundance, volume and cellular localisation of organelles between the life-cycle stages. The lysosome, a hydrolase-containing degradative compartment, is one organelle that changes in shape and content during the life-cycle of the parasite.

### 3.1 The lysosome: a dynamic compartment changing during the parasite’s life-cycle

**Fig. 3:**
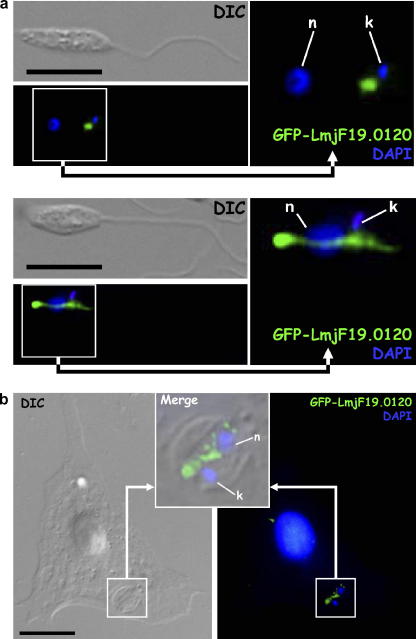
Labelling of the lysosomal compartment of *Leishmania major* using a GFP-fused syntaxin. GFP-LmjF19.0120 (green) was observed in a live procyclic promastigote (a, top), metacyclic promastigote (a, bottom) and amastigote within a mouse macrophage (b). Nuclear (n) and mitochondrial (kinetoplast, k) DNA were stained with DAPI (blue). The cells were visualised by differential interference contrast (DIC) and enlarged images are displayed on the right. Scale bar is 10 μm.

An ultrastructural study of *L. mexicana* promastigotes by Weise and colleagues (Weise et al., 2000) identified a post-Golgi tubo-vesicular compartment they termed the multivesicular tubule (MVT). It was later confirmed to be of lysosomal nature, as it contained several peptidases and was found to accumulate the endocytic tracer FM4-64 (Mullin et al., 2001). This lipophilic fluorescent dye initially binds to the cell surface of the parasites, before being endocytosed through the flagellar pocket and ultimately ends up in the lysosomal compartment. The use of FM4-64 has allowed the labelling of the MVT-lysosome in live *Leishmania* (Besteiro et al., 2006b; Mullin et al., 2001), and in addition other markers such as fluorescent lipid BODIPY-ceramide (Ghedin et al., 2001; Ilgoutz et al., 1999) and endocytosed fluorescent dextrans (Besteiro et al., 2006b; Ghedin et al., 2001) have also been used in this way. Overexpressed proteins, including green fluorescent protein (GFP)-tagged chimeras, have been also been found to accumulate in this compartment, probably to be degraded (Ghedin et al., 2001; Ilgoutz et al., 1999; Mullin et al., 2001; Weise et al., 2000). A more specific labelling was achieved expressing GFP-fused homologues of yeast vacuolar syntaxins (Besteiro et al., 2006a), which labelled a tubular compartment in promastigotes (Fig. 3a). The use of a microtubule-disrupting agent such as thioridazine (Ilgoutz et al., 1999) or ion-transporter inhibitors such as bafilomycin A1 (Mullin et al., 2001) causes the MVT to rapidly collapse into several large vesicles, suggesting that the tubule is normally under tension. The association with one or two microtubules and their potential role in maintaining the shape of the tubule have also been confirmed by EM observation (Mullin et al., 2001; Weise et al., 2000).

In live procyclic promastigotes, the lysosomal compartment is visualised as a large single vesicular structure at the anterior end of the cells after labelling with GFP-fused pro-domain of the trypanosome lysosomal cysteine peptidase cruzain (Huete-Pérez et al., 1999) or GFP-fused syntaxin LmjF19.0120 (Fig. 3a, top). These markers also label the MVT-lysosome in live metacyclic promastigotes (Fig. 3a, bottom). This suggests that the structure, content and, by inference, function of the lysosomal compartment evolves during differentiation from procyclic to metacyclic promastigote.

The differentiation of promastigotes into intracellular amastigotes is accompanied by further morphological changes of the lysosomal compartment. *Leishmania* amastigotes are characterised by the presence of a large membrane-bound compartment first identified in *L. mexicana* and termed “megasome” because of its large size. This structure displays lysosome-like properties such as an acidic pH and the presence of peptidases. The megasomes vary in aspect, size and numbers, depending on the species. For instance, morphometric and volume reconstruction studies showed that megasomes represent up to 15% of the total cell volume in lesion-derived amastigotes of *L. mexicana* (Coombs et al., 1986), whereas they represent ∼5% of the cell volume of *Leishmania amazonensis* amastigotes (Ueda-Nakamura et al., 2001). Interestingly, the size and aspect of megasomes might be linked to the nutrient requirements or the virulence potential of the amastigotes, as lesion-derived *L. amazonensis* amastigotes show smaller but more numerous megasomes than cultivated axenic amastigotes (Ueda-Nakamura et al., 2001). GFP-fused syntaxin LmjF19.0120 is distributed throughout the intracellular amastigote of *L. major* in numerous organelles (Fig. 2b), most likely megasomes, a localisation that is clearly different from the one observed in procyclic or metacyclic promastigotes. For a comprehensive review on the *Leishmania* lysosome see Waller and McConville, 2002.

### 3.2 Changes in lysosomal cysteine peptidase expression in the different life-cycle stages

The changes in the structure of the lysosomal compartment during cellular differentiation are accompanied by changes in its enzyme content. For instance, a consistent finding for megasomes from both lesion-derived and in vitro-transformed amastigotes from *L. mexicana* and *L. amazonensis* is the increase in the levels of expression of associated cysteine peptidases (Brooks et al., 2000, 2001; Ueda-Nakamura et al., 2002). During the differentiation within a macrophage of internalised *L. amazonensis* promastigotes into amastigotes, both the appearance of megasome-like structures and the increase in CPB-like cysteine peptidase activities appear within ∼24 h (Courret et al., 2001). Interestingly, megasome-like structures have not been observed in *Leishmania donovani* complex species, and amastigotes have relatively low cysteine peptidase activity (Mundodi et al., 2002, 2005). Thus the lysosomal compartment varies between *Leishmania* species. Similarly, changes in peptidase content occur in parallel with modification of the lysosomal compartment of *Leishmania* promastigotes during their in vitro growth. There is a correlation between the formation of the MVT-lysosome, which was shown by cellular fractionation studies to contain both serine peptidase and cysteine peptidases activity (Mullin et al., 2001), when procyclic promastigotes differentiate into metacyclic promastigotes, and an increase in overall proteolytic activity.

### 3.3 Role of lysosomal peptidases in nutrient acquisition

The energy metabolism of both promastigotes and amastigotes forms of *Leishmania* is not fully understood, but current data suggest that they differ significantly (Opperdoes and Coombs, 2007). Some secreted or intracellular peptidases could contribute in generating, together with exopeptidases, small peptides and amino acids that could feed into catabolic or biosynthetic pathways. However, the extent to which they are important in these ways has not in the main been addressed. Promastigotes in the midgut of the sandfly vector are initially bathed in a glucose-rich bloodmeal and subsequently sugars from nectar feeds (Gontijo et al., 1998). However, amino acids and notably proline are considered major substrates and these could be generated, in part at least, through the action of parasite-produced peptidases. Indeed, when cultivated in vitro promastigotes have been shown to secrete several peptidases that could have such a role, as could the surface-located leishmanolysin (Jaffe and Dwyer, 2003). Moreover, haemoglobin, available in the blood meal, has been shown to be internalised to a late endosomal/lysosomal compartment in promastigotes (Sengupta et al., 1999; Singh et al., 2003), and so it can be hypothesised that degradation by peptidases could result in the release of heme that is required by the parasite as it is unable to fully synthesise it (Opperdoes and Coombs, 2007). Peptidase may also be important in nutrient acquisition for the amastigote stage that multiplies within a parasitophorous vacuole and indeed some *Leishmania* cysteine peptidases have been found outside the parasite, perhaps the result of secretion (Duboise et al., 1994). These, perhaps, could degrade proteins within the vacuole, thus releasing peptides for the parasite to use. Probably more important are the intracellular peptidases that it is presumed are involved in the digestion of proteins after their uptake into the parasite. For instance, iron may be obtained by *Leishmania* through digestion by peptidases of internalised transferrin or lactoferrin. Transferrin has been reported to accumulate in the peptidase-rich megasomes (Borges et al., 1998), though other studies suggest that iron may be obtained through extracellular release from the chelate and subsequent uptake (Wilson et al., 2002). Although currently definitive data are lacking, it seems likely that intracellular peptidases in the lysosomal compartment play an important role in degrading proteins taken up by the parasite, and also in recycling cellular proteins delivered to the lysosomal compartment through the autophagic pathway.

## 4 Role of autophagy in protein turnover during differentiation of *Leishmania*

The lysosomal compartment is not only the end-point of the endocytic pathway, it is also associated with a crucial eukaryotic auto-degradative system – the autophagic pathway. Autophagy, the process of self-digestion by a cell which involves the action of degradative enzymes originating in the same cell, is important for protein and organelle degradation during cellular differentiation and also as a defense against starvation conditions (Reggiori and Klionsky, 2005). There are three main forms of autophagy: chaperone-mediated autophagy, microautophagy and macroautophagy. Chaperone-mediated autophagy is a secondary response to starvation or oxidative stress; it is a selective mechanism for the degradation of soluble cytosolic proteins in lysosomes and has been mainly documented in mammalian cells. Microautophagy is the least-characterised process, but is used to sequester part of the cytoplasm or nucleus by invagination or septation of the lysosomal membrane. Macroautophagy, which is the most prevalent and most studied form of autophagy, involves the formation of cytosolic double-membrane vesicles (termed autophagosomes) that sequester portions of the cytoplasm containing the organelles and macromolecules to be degraded (Reggiori and Klionsky, 2005). Autophagy has also been implicated as the mediator of type II programmed cell death (also known as autophagic cell death), which is quite different from apoptotic cell death (type I programmed cell death) that is caspase-dependent (Edinger and Thompson, 2004). There is some evidence that a form of programmed cell death occurs in *Leishmania* (Debrabant et al., 2003), which potentially could involve metacaspase (see Section 2.2) and apparently dying *Leishmania* exhibit enhanced autophagy (Williams et al., 2006). However, this may be an attempt to avoid death rather than a death process itself and so more substantive studies are required to understand whether autophagy has such a role in the parasite. Macroautophagy does, however, occur in *Leishmania* – being crucial for the differentiation between procyclic and metacyclic promastigotes and between metacyclic promastigotes and amastigotes (Besteiro et al., 2006b). The process requires peptidases for the digestion of the engulfed cellular material in the lysosomal compartment (Williams et al., 2006). Ubiquitin-mediated degradation of proteins via the proteasome is also likely to be important for differentiation (Paugam et al., 2003). Autophagy is induced by starvation, but whether this is physiologically important (such as for metacyclic promastigotes in the nutrient-deficient foregut of the sandfly (Bates, 2006)) remains to be determined, and the occurrence of many autophagosomes in dying *Leishmania* suggest that autophagy is also induced under some stresses, either as a survival strategy or, perhaps, as mentioned above, as a form of cell death (Debrabant et al., 2003).

### 4.1 The autophagic machinery of *L. major*

Genes coding for the proteins of the autophagic machinery are designated *ATG* and many have been identified in *L. major* (Herman et al., 2006; Rigden et al., 2005; Williams et al., 2006). The genesis of autophagosomal structures requires the activity of two protein conjugation systems. One involves ubiquitin-like protein ATG8, which is proteolytically processed by the Clan CA cysteine peptidase ATG4 prior to conjugation to phosphatidylethanolamine and then insertion into the autophagosomal membrane. A second involves a ubiquitin-like protein ATG12, covalently attached to ATG5 (Yorimitsu and Klionsky, 2005). The autophagosome delivers the internalised material to the lysosomal compartment for degradation. In mammals, but perhaps not in yeast, autophagosomes first fuse with endosomal vesicles.

**Fig. 4:**
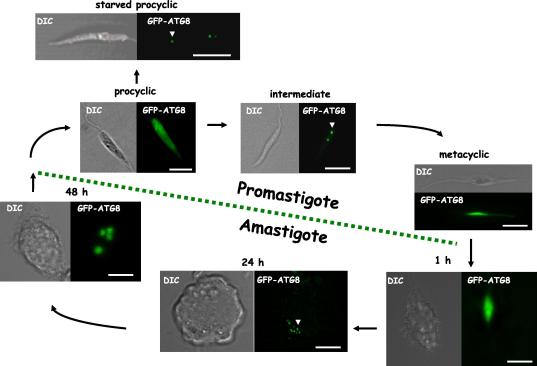
The distribution of GFP-ATG8 expressed in live *Leishmania* during its life-cycle. Examples of GFP-ATG8-labelled autophagosomes are indicated with arrows. GFP-ATG8 has a cytosolic distribution in replicating procyclic promastigotes, but some relocates to autophagosomes during metacyclogenesis or under starvation conditions. GFP-ATG8 is located in the MVT-lysosome in metacyclic promastigotes as the autophagosomes fuse with lysosomes to form autolysosomes. During differentiation from a metacyclic promastigote to an amastigote following infection of a macrophage, GFP-ATG8 has an initial cytosolic distribution (1 h p.i., one parasite visualised), associates with multiple autophagosomes at peak differentiation (24 h, one parasite visualised) and is subsequently found in the cytosol (48 h, two parasites visualised). Scale bar is 10 μm.

*Leishmania* possess two ATG4s, peptidases that hydrolyse the precursor form of ATG8 to its cytosolic form (ATG8-I) by exposing a C-terminal Gly residue. The exposed Gly is conjugated to phosphatidylethanolamine, catalysed by ATG7 (an E1-like enzyme) and ATG3 (an E2-like enzyme), to form membrane-bound ATG8-II. This localises to pre-autophagosomes and autophagosomes, which makes it an excellent autophagosomal marker and has been used as such experimentally. ATG8-II is subsequently deconjugated by ATG4 to release the protein from the surface (but not interior) of the autophagosome, permitting fusion of the autophagosome with endosomes/lysosomes. Expression of GFP-ATG8 in *Leishmania* has been used as a marker for autophagosomes for several reasons. Firstly, GFP-ATG8 localises to punctate structures (autophagosomes) that can be visualised by live cell fluorescent imaging and this has allowed monitoring of autophagy in individual cells in real time throughout the life-cycle (Fig. 4). Second, the lipidated form of GFP-ATG8 (GFP-ATG8-I, which is bound to autophagosomes) can be distinguished from the precursor and cleaved unlipidated forms (GFP-ATG8 and GFP-ATG8-I, respectively, both cytosolic) by gel electrophoresis – formation of lipidated ATG8 being the accepted marker for autophagosome formation in mammalian systems. Third, no phenotype can be detected in cells expressing GFP-ATG8, suggesting the marker itself does not significantly affect the parasite. There are at least three families of ATG8-related proteins in mammals (LC3, GABARAP and GATE16) each of which has a number of sub-families (e.g. three LC3 genes have been identified). *Leishmania major* also has a large number of *ATG8*-related genes, each of which has subfamilies (2 × *ATG8* [most similar to mammalian LC3], 3× *ATG8A*, 9× *ATG8B* and 13× *ATG8C*), which are conserved across different *Leishmania* species. The presence of such a large gene family of *ATG8*-related genes in *L. major* implies *Leishmania*-specific functions, possibly involving autophagy. The two isoforms of the ATG4 cysteine peptidase (ATG4.1 and ATG 4.2) in *L. major* may well selectively cleave the members of the ATG8 families involved in different autophagic pathways with specific function and stimulus.

### 4.2 Autophagosome biogenesis and degradation

Recent reports suggest that lipid from pre-existing endoplasmic reticulum (ER) flows to the early secretory pathway and this initiates the formation of the pre-autophagosomal membrane (Mijaljica et al., 2006). The involvement of the ER and Golgi for the formation of autophagosomes in *Leishmania* has also been suggested, mainly from ultrastructural observations. Autophagosome-like structures have been described in a variety of *Leishmania* species with either double- or a single-membrane with a diameter ranging from 0.6 to 1.5 μm (Besteiro et al., 2006b; Ledezma et al., 2002; Santa-Rita et al., 2004; Williams et al., 2006). Their luminal contents appeared to be cytosolic proteins (Williams et al., 2006), multi-vesicular bodies (Vannier-Santos and Lins, 2001) and even acidocalcisomes (Vannier-Santos and Lins, 2001). The possibility that glycosomes are also degraded in autophagosome is likely (Herman et al., 2006; Michels et al., 2005), as pexophagy is a well established mechanism for turnover of peroxisomes in yeast.

Fusion of the autophagosomes with the lysosomes in yeast and mammalian cells requires acidification, vacuolar ATPases, the endocytic pathway, soluble *N*-ethylmaleimide-sensitive factor adaptor proteins receptors (SNARE) molecules and a functional microtubular network (Eskelinen, 2005). There is some evidence that such are also involved in *Leishmania* (Besteiro et al., 2006b; Williams et al., 2006). Autophagosomes are eventually delivered to the lysosome and are degraded by a repertoire of hydrolytic enzymes. In *S. cerevisiae*, the aspartic peptidase PEP4 and the serine peptidase PBR1 are particularly important. However, *Leishmania* lacks orthologues of these enzymes and instead the cysteine peptidases CPA and CPB, appear to be key to autophagosome degradation in the MVT-lysosome (Williams et al., 2006). The aspartic peptidase Presenilin 1 has been reported to play a role in mammalian cell autophagosome maturation and fusion with lysosomes and protein turnover by macropautophagy is almost totally absent in presenilin 1 knockout mice (Shen and Kelleher, 2007). The *L. major* presenilin 1 orthologue contains many of the domains known to be important for the activity of the membrane associated aspartic peptidase, so a role for the enzyme in macroautophagy is possible.

### 4.3 Role of autophagy in intracellular degradation and differentiation

During metacyclogenesis and transformation into amastigotes an increase in the abundance of autophagosomes (Besteiro et al., 2006b; Williams et al., 2006) and protein degradation (Alves et al., 2005) have been observed. The coordinated up-regulation of both the autophagic and proteolytic machineries appears to be instrumental in allowing cellular remodelling during differentiation. Interfering with this process through impairment of lysosomal function with cysteine peptidase inhibitors or the creation of mutant parasites deficient in cysteine peptidases CBA and CPB results in promastigotes that are apparently unaffected in their ability to grow and multiply in nutrient-rich medium in vitro, presumably as they can tolerate the accumulation of damaged and non-recycled constituents, although they have greater numbers of autophagosomes-containing multi-vesicular bodies (Selzer et al., 1999; Williams et al., 2006). The multiple cell divisions that occur during this growth are associated with intensive synthesis of new biological structures, which presumably dilute the effects of damaged cellular constituents and so facilitate survival. However the perturbation interrupts the cell remodelling accompanying metacyclogenesis such that the process is largely abrogated, and similarly differentiation to amastigotes is greatly hindered. Thus the current evidence is that protein turnover in *Leishmania* is crucial for successful differentiation of the parasite.

## 5 Conclusion

*Leishmania* contains a vast repertoire of proteolytic enzymes. The output from the *L. major* genome project allowed the identification of many of them, and some functional studies have already showed that several are important for the development of the parasite and the transition between its different developmental forms. However, much remains to be done to assign a function to most of the enzymes. One aspect yet to be fully considered is that peptidases may perform functions unrelated to their enzymatic activity. Instead, the structure of particular domains may be the key factor mediating their effect. Irrespective of this, association with a specific compartment in order to perform a specific cellular function is probably the norm. The data obtained so far on the degradative compartments of *Leishmania*, notably the lysosome but also the autophagic pathway, show that they undergo remarkable morphological changes and that these appear to correlate, not surprisingly, with changes in ability to turnover proteins. The context is now known, the next stages are to analyse more fully the roles of the various peptidases in the processes that are key to the parasites’ growth and survival.

## Figures and Tables

**Table 1:** Estimate of the distribution of peptidases in *Leishmania major*, *Trypanosoma brucei* and *Trypanosoma cruzi* (Ivens et al., 2005), *Plasmodium falciparum* (Wu et al., 2003), Human (Puente et al., 2003) and Yeast (http://merops.sanger.ac.uk/)

Species	Catalytic type					
	Total	Aspartic	Cysteine	Metallo	Serine	Threonine
*L. major*	154^a^	2	62	55	13	21
*T. brucei*	144	2	57	51	18	16
*T. cruzi*	397	3	98	241	30	25
*P. falciparum*	92	10	33	20	16	13
Human	561	21	148	186	178	28
Yeast	100	15	29	36	17	3

a Includes one peptidase of unknown catalytic type (family U48).

## References

[bib1] Alves C.R., Corte-Real S., Bourguignon S.C., Chaves C.S., Saraiva E.M. (2005). *Leishmania amazonensis*: early proteinase activities during promastigote-amastigote differentiation *in vitro*. Exp. Parasitol..

[bib2] Barak E., Amin-Spector S., Gerliak E., Goyard S., Holland N., Zilberstein D. (2005). Differentiation of *Leishmania donovani* in host-free system: analysis of signal perception and response. Mol. Biochem. Parasitol..

[bib3] Bateman A., Rawlings N.D. (2003). The CHAP domain: a large family of amidases including GSP amidase and peptidoglycan hydrolases. Trends Biochem. Sci..

[bib4] Bates P.A. (2006). Housekeeping by *Leishmania*. Trends Parasitol..

[bib5] Bates P.A., Rogers M.E. (2004). New insights into the developmental biology and transmission mechanisms of *Leishmania*. Curr. Mol. Med..

[bib6] Besteiro S., Coombs G.H., Mottram J.C. (2004). A potential role for ICP, a leishmanial inhibitor of cysteine peptidases, in the interaction between host and parasite. Mol. Microbiol..

[bib7] Besteiro S., Coombs G.H., Mottram J.C. (2006). The SNARE protein family of *Leishmania major*. BMC genomics.

[bib8] Besteiro S., Williams R.A.M., Morrison L.S., Coombs G.H., Mottram J.C. (2006). Endosome sorting and autophagy are essential for differentiation and virulence of *Leishmania major*. J. Biol. Chem..

[bib9] Borges V.M., Marcos A.V., Wanderley d.S. (1998). Subverted transferrin trafficking in *Leishmania*-infected macrophages. Parasitol. Res..

[bib10] Brinkmann V., Reichard U., Goosmann C., Fauler B., Uhlemann Y., Weiss D.S., Weinrauch Y., Zychlinsky A. (2004). Neutrophil extracellular traps kill bacteria. Science.

[bib11] Brooks D.R., Denise H., Westrop G.D., Coombs G.H., Mottram J.C. (2001). The stage-regulated expression of *Leishmania mexicana* CPB cysteine proteases is mediated by an intercistronic sequence element. J. Biol. Chem..

[bib12] Brooks D.R., Tetley L., Coombs G.H., Mottram J.C. (2000). Processing and trafficking of cysteine proteases in *Leishmania mexicana*. J. Cell Sci..

[bib13] Caler E.V., De Avalos S.V., Haynes P.A., Andrews N.W., Burleigh B.A. (1998). Oligopeptidase B-dependent signaling mediates host cell invasion by *Trypanosoma cruzi*. EMBO J..

[bib14] Campbell D.A., Thomas S., Sturm N.R. (2003). Transcription in kinetoplastid protozoa: why be normal?. Microbes Infect..

[bib15] Chung C.H., Ives H.E., Almeda S., Goldberg A.L. (1983). Purification from *Escherichia coli* of a periplasmic protein that is a potent inhibitor of pancreatic proteases. J. Biol. Chem..

[bib16] Coombs G.H., Goldberg D.E., Klemba M.W., Berry C., Kay J., Mottram J.C. (2001). Aspartic proteases of *Plasmodium falciparum* and other parasitic protozoa as drug targets. Trends Parasitol..

[bib17] Coombs G.H., Tetley L., Moss V.A., Vickerman K. (1986). Three dimensional structure of the *Leishmania* amastigote as revealed by computer-aided reconstruction from serial sections. Parasitology.

[bib18] Courret N., Frehel C., Prina E., Lang T., Antoine J.C. (2001). Kinetics of the intracellular differentiation of *Leishmania amazonensis* and internalization of host MHC molecules by the intermediate parasite stages. Parasitology.

[bib19] Debrabant A., Lee N., Bertholet S., Duncan R., Nakhasi H.L. (2003). Programmed cell death in trypanosomatids and other unicellular organisms. Int. J. Parasitol..

[bib20] del Pozo O., Lam E. (1998). Caspases and programmed cell death in the hypersensitive response of plants to pathogens. Curr. Biol..

[bib21] Duboise S.M., Vannier-Santos M.A., Costa-Pinto D., Rivas L., Pan A.A., Traub-Cseko Y.M., de Souza W., McMahon-Pratt D. (1994). The biosynthesis, processing, and immunolocalization of *Leishmania pifanoi* amastigote cysteine proteinases. Mol. Biochem. Parasitol..

[bib22] Edinger A.L., Thompson C.B. (2004). Death by design: apoptosis, necrosis and autophagy. Curr. Opin. Cell Biol..

[bib23] Eggers C.T., Murray I.A., Delmar V.A., Day A.G., Craik C.S. (2004). The periplasmic serine protease inhibitor ecotin protects bacteria against neutrophil elastase. Biochem. J..

[bib24] Eschenlauer S.C., Coombs G.H., Mottram J.C. (2006). *PFPI*-like genes are expressed in *Leishmania major* but are pseudogenes in other *Leishmania* species. FEMS Microbiol. Lett..

[bib25] Eskelinen E.L. (2005). Maturation of autophagic vacuoles in mammalian cells. Autophagy.

[bib26] Ghedin E., Debrabant A., Engel J.C., Dwyer D.M. (2001). Secretory and endocytic pathways converge in a dynamic endosomal system in a primitive protozoan. Traffic.

[bib27] Gontijo N.F., Almeida-Silva S., Mares-Guia M.L., Williams P., Melo M.N. (1998). *Lutzomyia longipalpis*: pH in the gut, digestive glycosidases, and some speculations upon *Leishmania* development. Exp. Parasitol..

[bib28] Gonzales I.J., Desponds C., Schaff C., Mottram J.C., Fasel N. (2007). *Leishmania major* metacaspase can replace yeast metacaspase in programmed cell death and has arginine-specific cysteine peptidase activity. Int. J. Parasitol..

[bib29] Gossage S.M., Rogers M.E., Bates P.A. (2003). Two separate growth phases during the development of *Leishmania* in sand flies: implications for understanding the life cycle. Int. J. Parasitol..

[bib30] Grellier P., Vendeville S., Joyeau R., Bastos I.M.D., Drobecq H., Frappier F., Teixeira A.R.L., Schrével J., Davioud-Charvet E., Sergheraert C., Santana J.M. (2001). *Trypanosoma cruzi* prolyl oligopeptidase Tc80 is involved in nonphagocytic mammalian cell invasion by trypomastigotes. J. Biol. Chem..

[bib31] Herker E., Jungwirth H., Lehmann K.A., Maldener C., Frohlich K.U., Wissing S., Buttner S., Fehr M., Sigrist S., Madeo F. (2004). Chronological aging leads to apoptosis in yeast. J. Cell. Biol..

[bib32] Herman M., Gillies S., Michels P.A., Rigden D.J. (2006). Autophagy and related processes in trypanosomatids: insights from genomic and bioinformatic analyses. Autophagy.

[bib33] Hide M., Bras-Goncalves R., Banuls A.L. (2007). Specific cpb copies within the *Leishmania donovani* complex: evolutionary interpretations and potential clinical implications in humans. Parasitology.

[bib34] Hilley J.D., Zawadzki J., McConville M.J., Coombs G.H., Mottram J.C. (2000). *Leishmania mexicana* mutants lacking glycosylphosphatidyl (GPI): protein transamidase provide insights into the biosynthesis and functions of GPI-anchored proteins. Mol. Biol. Cell.

[bib35] Holzer T.R., McMaster W.R., Forney J.D. (2006). Expression profiling by whole-genome interspecies microarray hybridization reveals differential gene expression in procyclic promastigotes, lesion-derived amastigotes, and axenic amastigotes in *Leishmania mexicana*. Mol. Biochem. Parasitol..

[bib36] Huete-Pérez J.A., Engel J.C., Brinen L.S., Mottram J.C., McKerrow J.H. (1999). Protease trafficking in two primitive eukaryotes is mediated by a prodomain protein motif. J. Biol. Chem..

[bib37] Ilgoutz S.C., Mullin K.A., Southwell B.R., McConville M.J. (1999). Glycosylphosphatidylinositol biosynthetic enzymes are localized to a stable tubular subcompartment of the endoplasmic reticulum in *Leishmania mexicana*. EMBO J..

[bib38] Ivens, A.C. et al., 2005. The genome of the kinetoplastid parasite, *Leishmania major*. Science 309, 436–442. 10.1126/science.1112680 PMC1470643 16020728

[bib39] Jaffe C.L., Dwyer D.M. (2003). Extracellular release of the surface metalloprotease, gp63, from *Leishmania* and insect trypanosomatids. Parasitol. Res..

[bib40] Ledezma E., Jorquera A., Bendezu H., Vivas J., Perez G. (2002). Antiproliferative and leishmanicidal effect of ajoene on various *Leishmania* species: ultrastructural study. Parasitol. Res..

[bib41] Madeo F., Herker E., Maldener C., Wissing S., Lachelt S., Herlan M., Fehr M., Lauber K., Sigrist S.J., Wesselborg S., Frohlich K.U. (2002). A caspase-related protease regulates apoptosis in yeast. Mol. Cell..

[bib42] McNicoll F., Drummelsmith J., Muller M., Madore E., Boilard N., Ouellette M., Papadopoulou B. (2006). A combined proteomic and transcriptomic approach to the study of stage differentiation in *Leishmania infantum*. Proteomics.

[bib43] Michels P.A., Moyersoen J., Krazy H., Galland N., Herman M., Hannaert V. (2005). Peroxisomes, glyoxysomes and glycosomes. Mol. Membr. Biol..

[bib44] Mijaljica D., Prescott M., Devenish R.J. (2006). Endoplasmic reticulum and golgi complex: contributions to, and turnover by, autophagy. Traffic.

[bib45] Morty R.E., Pelle R., Vadasz I., Uzcanga G.L., Seeger W., Bubis J. (2005). Oligopeptidase B from *Trypanosoma evansi*. A parasite peptidase that inactivates atrial natriuretic factor in the bloodstream of infected hosts. J. Biol. Chem..

[bib46] Mottram J.C., Coombs G.H., Alexander J. (2004). Cysteine peptidases as virulence factors of *Leishmania*. Curr. Opin. Microbiol..

[bib47] Mottram J.C., Helms M.J., Coombs G.H., Sajid M. (2003). Clan CD cysteine peptidases of parasitic protozoa. Trends Parasitol..

[bib48] Mullin K.A., Foth B.J., Ilgoutz S.C., Callaghan J.M., Zawadzki J.L., McFadden G.I., McConville M.J. (2001). Regulated degradation of an endoplasmic reticulum membrane protein in a tubular lysosome in *Leishmania mexicana*. Mol. Biol. Cell.

[bib49] Mundodi V., Kucknoor A.S., Gedamu L. (2005). Role of *Leishmania* (*Leishmania*) *chagasi* amastigote cysteine protease in intracellular parasite survival: studies by gene disruption and antisense mRNA inhibition. BMC. Mol. Biol..

[bib50] Mundodi V., Somanna A., Farrell P.J., Gedamu L. (2002). Genomic organization and functional expression of differentially regulated *cysteine protease* genes of *Leishmania donovani* complex. Gene.

[bib51] Opperdoes F.R., Coombs G.H. (2007). Metabolism of *Leishmania*: proven and predicted. Trends Parasitol..

[bib52] Parussini F., García M., Mucci J., Agüero F., Sánchez D., Hellman U., Åslund L., Cazzulo J.J. (2003). Characterization of a lysosomal serine carboxypeptidase from *Trypanosoma cruzi*. Mol. Biochem. Parasitol..

[bib53] Paugam A., Bulteau A.L., Dupouy-Camet J., Creuzet C., Friguet B. (2003). Characterization and role of protozoan parasite proteasomes. Trends Parasitol..

[bib54] Puente X.S., Sanchez L.M., Overall C.M., Lopez-Otin C. (2003). Human and mouse proteases: a comparative genomics approach. Nat. Rev. Genet..

[bib55] Ramalho-Ortigao J.M., Kamhawi S., Rowton E.D., Ribeiro J.M.C., Valenzuela J.G. (2003). Cloning and characterization of trypsin- and chymotrypsin-like proteases from the midgut of the sand fly vector *Phlebotomus papatasi*. Insect Biochem. Mol. Biol..

[bib56] Rawlings N.D., Tolle D.P., Barrett A.J. (2004). Evolutionary families of peptidase inhibitors. Biochem. J..

[bib57] Rawlings N.D., Tolle D.P., Barrett A.J. (2004). MEROPS: the peptidase database. Nucleic Acids Res..

[bib58] Reggiori F., Klionsky D.J. (2005). Autophagosomes: biogenesis from scratch?. Curr. Opin. Cell Biol..

[bib59] Ribeiro-Gomes F.L., Otero A.C., Gomes N.A., Moniz-de-Souza M.C., Cysne-Finkelstein L., Arnholdt A.C., Calich V.L., Coutinho S.G., Lopes M.F., DosReis G.A. (2004). Macrophage interactions with neutrophils regulate *Leishmania major* infection. J. Immunol..

[bib60] Rigden D.J., Herman M., Gillies S., Michels P.A. (2005). Implications of a genomic search for autophagy-related genes in trypanosomatids. Biochem. Soc. Trans..

[bib61] Rigden D.J., Mosolov V.V., Galperin M.Y. (2002). Sequence conservation in the chagasin family suggests a common trend in cysteine proteinase binding by unrelated protein inhibitors. Protein Sci..

[bib62] Robertson C.D. (1999). The *Leishmania mexicana* proteasome. Mol. Biochem. Parasitol..

[bib63] Sanderson S.J., Westrop G.D., Scharfstein J., Mottram J.C., Coombs G.H. (2003). Functional conservation of a natural cysteine peptidase inhibitor in protozoan and bacterial pathogens. FEBS Lett..

[bib64] Santa-Rita R.M., Henriques-Pons A., Barbosa H.S., De Castro S.L. (2004). Effect of the lysophospholipid analogues edelfosine, ilmofosine and miltefosine against *Leishmania amazonensis*. J. Antimicrob. Chemother..

[bib65] Saxena A., Lahav T., Holland N., Aggarwal G., Anupama A., Huang Y., Volpin H., Myler P.J., Zilberstein D. (2007). Analysis of the *Leishmania donovani* transcriptome reveals an ordered progression of transient and permanent changes in gene expression during differentiation. Mol. Biochem. Parasitol..

[bib66] Schaeffer M., de Miranda A., Mottram J.C., Coombs G.H. (2006). Differentiation of *Leishmania major* is impaired by over-expression of pyroglutamyl peptidase I. Mol. Biochem. Parasitol..

[bib67] Selzer P.M., Pingel S., Hsieh I., Ugele B., Chan V.J., Engel J.C., Bogyo M., Russell D.G., Sakanari J.A., McKerrow J.H. (1999). Cysteine protease inhibitors as chemotherapy: lessons from a parasite target. Proc. Natl. Acad. Sci. USA.

[bib68] Sengupta S., Tripathi J., Tandon R., Raje M., Roy R.P., Basu S.K., Mukhopadhyay A. (1999). Hemoglobin endocytosis in *Leishmania* is mediated through a 46-kDa protein located in the flagellar pocket. J. Biol. Chem..

[bib69] Shen J., Kelleher R.J. (2007). The presenilin hypothesis of Alzheimer’s disease: evidence for a loss-of-function pathogenic mechanism. Proc. Natl. Acad. Sci. USA.

[bib70] Singh S.B., Tandon R., Krishnamurthy G., Vikram R., Sharma N., Basu S.K., Mukhopadhyay A. (2003). Rab5-mediated endosome-endosome fusion regulates hemoglobin endocytosis in *Leishmania donovani*. EMBO J..

[bib71] Smith B.O., Picken N.C., Westrop G.D., Bromek K., Mottram J.C., Coombs G.H. (2006). The structure of *Leishmania mexicana* ICP provides evidence for convergent evolution of cysteine peptidase inhibitors. J. Biol. Chem..

[bib72] Ueda-Nakamura T., Attias M., de Souza W. (2001). Megasome biogenesis in *Leishmania amazonensis*: a morphometric and cytochemical study. Parasitol. Res..

[bib73] Ueda-Nakamura T., da Conceicao Rocha Sampaio M., Cunha e Silva T., Traub-Cseko Y.M., de Souza W. (2002). Expression and processing of megasome cysteine proteinases during *Leishmania amazonensis* differentiation. Parasitol. Res..

[bib74] Urban S. (2006). Rhomboid proteins: conserved membrane proteases with divergent biological functions. Genes Dev..

[bib75] Uren A.G., O’Rourke K., Aravind L.A., Pisabarro M.T., Seshagiri S., Koonin E.V., Dixit V.M. (2000). Identification of paracaspases and metacaspases: two ancient families of caspase-like proteins, one of which plays a key role in MALT lymphoma. Mol. Cell..

[bib76] Van Zandbergen G., Klinger M., Mueller A., Dannenberg S., Gebert A., Solbach W., Laskay T. (2004). Cutting edge: neutrophil granulocyte serves as a vector for *Leishmania* entry into macrophages. J. Immunol..

[bib77] Vannier-Santos M.A., Lins U. (2001). Cytochemical techniques and energy-filtering transmission electron microscopy applied to the study of parasitic protozoa. Biol. Proc. Online.

[bib78] Walker J., Vasquez J.J., Gomez M.A., Drummelsmith J., Burchmore R., Girard I., Ouellette M. (2006). Identification of developmentally-regulated proteins in *Leishmania panamensis* by proteome profiling of promastigotes and axenic amastigotes. Mol. Biochem. Parasitol..

[bib79] Waller R.F., McConville M.J. (2002). Developmental changes in lysosome morphology and function *Leishmania* parasites. Int. J. Parasitol..

[bib80] Weise F., Stierhof Y.D., Kühn C., Wiese M., Overath P. (2000). Distribution of GPI-anchored proteins in the protozoan parasite *Leishmania*, based on an improved ultrastructural description using high-pressure frozen cells. J. Cell Sci..

[bib81] Williams R.A.M., Tetley L., Mottram J.C., Coombs G.H. (2006). Cysteine peptidases CPA and CPB are vital for autophagy and differentiation in *Leishmania mexicana*. Mol. Microbiol..

[bib82] Wilson M.E., Lewis T.S., Miller M.A., McCormick M.L., Britigan B.E. (2002). *Leishmania chagasi*: uptake of iron bound to lactoferrin or transferrin requires an iron reductase. Exp. Parasitol..

[bib83] Wu Y., Wang X., Liu X., Wang Y. (2003). Data-mining approaches reveal hidden families of proteases in the genome of malaria parasite. Genome Res..

[bib84] Xia W., Wolfe M.S. (2003). Intramembrane proteolysis by presenilin and presenilin-like proteases. J. Cell. Sci..

[bib85] Yan J., Cheng Q., Li C.B., Aksoy S. (2001). Molecular characterization of two serine proteases expressed in gut tissue of the African trypanosome vector, *Glossina morsitans morsitans*. Insect. Mol. Biol..

[bib86] Yanagida M. (2005). Basic mechanism of eukaryotic chromosome segregation. Philos. Trans. Roy. Soc. Lond. B Biol. Sci..

[bib87] Yao C.Q., Donelson J.E., Wilson M.E. (2003). The major surface protease (MSP or GP63) of *Leishmania* species – biosynthesis, regulation of expression, and function. Mol. Biochem. Parasitol..

[bib88] Yorimitsu T., Klionsky D.J. (2005). Autophagy: molecular machinery for self-eating. Cell Death Differ..

